# Plastid Genome Comparative and Phylogenetic Analyses of the Key Genera in Fagaceae: Highlighting the Effect of Codon Composition Bias in Phylogenetic Inference

**DOI:** 10.3389/fpls.2018.00082

**Published:** 2018-02-01

**Authors:** Yanci Yang, Juan Zhu, Li Feng, Tao Zhou, Guoqing Bai, Jia Yang, Guifang Zhao

**Affiliations:** ^1^Key Laboratory of Resource Biology and Biotechnology in Western China (Ministry of Education), College of Life Sciences, Northwest University, Xi'an, China; ^2^Middle School of Xi'an Electronic Science and Technology, Xi'an, China; ^3^School of Pharmacy, Xi'an Jiaotong University, Xi'an, China; ^4^Shaanxi Engineering Research Centre for Conservation and Utilization of Botanical Resources, Xi'an Botanical Garden of Shaanxi Province, Xi'an, China

**Keywords:** Fagaceae, plastid genome, topological incongruence, codon composition bias, phylogenomics

## Abstract

Fagaceae is one of the largest and economically important taxa within Fagales. Considering the incongruence among inferences from plastid and nuclear genes in the previous Fagaceae phylogeny studies, we assess the performance of plastid phylogenomics in this complex family. We sequenced and assembled four complete plastid genomes (*Fagus engleriana, Quercus spinosa, Quercus aquifolioides*, and *Quercus glauca*) using reference-guided assembly approach. All of the other 12 published plastid genomes in Fagaceae were retrieved for genomic analyses (including repeats, sequence divergence and codon usage) and phylogenetic inference. The genomic analyses reveal that plastid genomes in Fagaceae are conserved. Comparing the phylogenetic relationships of the key genera in Fagaceae inferred from different codon positions and gene function datasets, we found that the first two codon sites dataset recovered nearly all relationships and received high support. Thus, the result suggested that codon composition bias had great influence on Fagaceae phylogenetic inference. Our study not only provides basic understanding of Fagaceae plastid genomes, but also illuminates the effectiveness of plastid phylogenomics in resolving relationships of this intractable family.

## Introduction

Due to the rapid development of next-generation sequencing (NGS) technology, genomic data have been increasingly used to explore plant phylogeny. With respect to genomic complexity, sequencing cost, analysis methods and the degree of recombination of different genomes (organelle genomes and nuclear genome), the plastid genome presents obvious advantages (e.g., generally recombination-free, uniparental inheritance, highly conserved structure) (Birky et al., 1983; Jansen and Ruhlman, 2012). Recently, the use of plastid genomes in plant phylogenetic analyses is expanding and great progress has been achieved (Jansen et al., 2007; Moore et al., 2007, 2010; Parks et al., 2009; Barrett et al., 2013, 2016; Nikiforova et al., 2013; Ma et al., 2014; Carbonell-Caballero et al., 2015). It is widely accepted that plastids are derived from an endosymbiotic event (Keeling, 2010, and references therein). Most angiosperm plastid genomes have a typical quadripartite structure, with two copies of inverted repeat regions (IR) separating the small and large single copy regions (SSC and LSC, respectively) (Jansen et al., 2005; Jansen and Ruhlman, 2012). Although the structure of the plastid genome is generally highly conserved, different levels of genomic upheaval (such as gene or IR losses, large-scale rearrangements) have been detected in Campanulaceae, Fabaceae, Geraniaceae, Oleaceae and many other families (Cosner et al., 2004; Lee et al., 2007; Cai et al., 2008; Guisinger et al., 2010, 2011; Martin et al., 2014).

Fagaceae is a diverse and ecologically dominant group throughout the Northern Hemisphere, which consists of 10 genera and ca. 900 species (Manos et al., 2001, 2008; Oh and Manos, 2008). In Fagaceae, the genus *Quercus* is species-rich (approximately 500 species worldwide) and has received substantial attention in phylogeny and biogeography studies compared with other genera (e.g., Manos et al., 1999; Cavender-Bares et al., 2004; Zeng et al., 2011; Gugger and Cavender-Bares, 2013). With many meaningful evolutionary topics to explore, Fagaceae is among the best studied woody plant families. For example, extensive hybridization resulting in perplexing taxonomy; rich fossil record for macroevolutionary studies; highly disparate fruit forms for studies of dispersal mode; and phylogenetic relationships of this species-rich family.

Previously, molecular phylogenies obtained from nuclear data appeared more plausible than those from plastid data (Manos et al., 2001; Denk and Grimm, 2010; Hubert et al., 2014), owing to their congruence with morphological evidence, including the fossil record (e.g., Denk and Grimm, 2009; Grímsson et al., 2015, 2016). Notably, combining two nuclear loci (ITS and *CRC*) with data from three plastid regions (*trnK*-*matk*/*trnK, atpB*-*rbcL* and *ndhF*) failed to resolve all oaks as one clade (Manos et al., 2008). Moreover, the same phenomenon was observed in Simeone et al. (2016) and Vitelli et al. (2017) that only used three plastid markers (both used plastid regions: *rbcL, trnK*/*matK*, and *trnH*-*psbA*). However, when using two nuclear loci (ITS and *CRC*) alone, they clarified the relationships of Fagaceae, in particular, oaks were supported as monophyletic (Oh and Manos, 2008). The phenomenon that plastid data and nuclear data generate conflicting (incongruent) phylogenies has also been observed in other plant groups, such as Senecioneae, *Helichrysum* and Neotropical Catasetinae (Pelser et al., 2010; Galbany-Casals et al., 2014; Pérez-Escobar et al., 2015). Topological incongruence may result from different genetic backgrounds (maternal or biparental inheritance) and substitution rates of plastid and nucleus (Tepe et al., 2011). Moreover, biological processes, such as chloroplast capture (by hybridization or introgression) and incomplete lineage sorting may also be responsible for the phenomenon (Stegemann et al., 2012; Pérez-Escobar et al., 2015).

In general, improvements in tree resolution of Fagaceae had been offered in the previous molecular studies. Nuclear markers, used in Fagaceae phylogeny inference, yielded relatively low support for the monophyletic genus *Quercus* (MP and ML bootstrap support values were 60 and 52, respectively) (Oh and Manos, 2008). Considering the performances of the few molecular markers in Fagaceae phylogenetic inferences and the ability of plastid phylogenomics (high resolution and strong support) in the earlier studies, we explore whether plastid genome-scale data have the ability to infer strongly supported phylogenetic relationships for Fagaceae, especially for the monophyletic genus *Quercus*.

## Materials and methods

### Taxon sampling and plant material

In total, 16 plastid genomes belonging to the key genera of Fagaceae are analyzed in this study, including four newly generated plastid genomes (*F. engleriana, Q. glauca, Q. spinosa*, and *Q. aquifolioides*) and all of the published plastid genomes in Fagaceae. The other 12 species are *Trigonobalanus doichangensis, Quercus rubra* (Alexander and Woeste, 2014), *Quercus baronii* (Yang et al., 2017), *Quercus aliena, Quercus aliena* var. *acuteserrata, Quercus variabilis, Quercus dolicholepis* (Yang et al., 2016), *Quercus edithiae, Castanopsis echinocarpa, Lithocarpus balansae, Castanea mollissima* (Jansen et al., 2011), and *Castanea pumila* var. *pumila* (Dane et al., 2015). The collecting and GenBank accession information for the analyzed taxa are listed in Table 1.

**Table 1:** Accessions in this study with taxonomic, collection locality, Illumina read, and coverage information.

**Species**	**Genus**	**Collection locality**	**GenBank number**	**Assembly reads**	**Mean coverage**
*Quercus rubra*	Group *Lobatae, Quercus*	/	JX970937	/	/
*Quercus aliena*	Group *Quercus, Quercus*	/	KU240007	/	/
*Quercus aliena* var. *acuteserrata*	Group *Quercus, Quercus*	/	KU240008	/	/
*Quercus baronii*	Group *Ilex, Quercus*	/	KT963087	/	/
*Quercus dolicholepis*	Group *Ilex, Quercus*	/	KU240010	/	/
*Quercus variabilis*	Group *Cerris, Quercus*	/	KU240009	/	/
*Quercus aquifolioides*	Group *Ilex, Quercus*	Panzhihua, Sichuan, China	KX911971	788,550	616x
*Quercus spinosa*	Group *Ilex, Quercus*	Dali, Yunnan, China	KX911972	766,767	591x
*Quercus glauca*	Group *Cyclobalanopsis, Quercus*	Chenshan Botanical Garden, Shanghai, China	KX852399	427,422	329 x
*Quercus edithiae*	Group *Cyclobalanopsis, Quercus*	/	KU382355	/	/
*Castanea mollissima*	*Castanea*	/	HQ336406	/	/
*Castanea pumila* var. *pumila*	*Castanea*	/	KM360048	/	/
*Castanopsis echinocarpa*	*Castanopsis*	/	KJ001129	/	/
*Lithocarpus balansae*	*Lithocarpus*	/	KP299291	/	/
*Trigonobalanus doichangensis*	*Trigonobalanus*	/	KF990556	/	/
*Fagus engleriana*	*Fagus*	Wuhan Botanical Garden, Wuhan, China	KX852398	362,613	281x

### DNA extraction, illumina sequencing, assembly, and annotation

Total genomic DNA was extracted for the four species from silica-dried leaf material following the modified CTAB method (Doyle, 1987). The paired-end (PE) library was constructed using TruSeq DNA sample preparation kits. Sequencing was completed on an Illumina Hiseq 2500 platform with the average read length of 125 bp, yielding at least 2 GB clean data for each species. All of the above work were conducted by Biomarker Technologies Inc. (Beijing, China). Firstly, all of the raw reads were trimmed using NGS QC Toolkit_v.2.3.3 with the default parameters set (Patel and Jain, 2012). Reference-guided assembly was then used to reconstruct the plastid genomes with the programs MIRA 4.0.2 (Chevreux et al., 2004) and MITObim v1.7 (Hahn et al., 2013). In the process, plastid genomes of *Q. rubra* (JX970937), *Q. aliena* (KU240007), and *C. mollissima* (HQ336406) were used as reference genomes. The complete plastid genomes were annotated using the program DOGMA (Wyman et al., 2004), and then manually corrected by comparing them with the complete plastid genomes of the other published Fagaceae species in GENEIOUS R8 (Biomatters Ltd., Auckland, New Zealand).

### Codon usage bias analysis

The protein-coding genes (CDS) were extracted from plastid genomes with the following constraints: (1) the presence of proper initial (ATG) and termination codons (TAA, TGA and TAG); (2) CDS length was greater than 300 bp to avoid sampling bias (Wright, 1990). Finally, 53 common CDS for each plastome were analyzed.

The GC content of the complete plastid genomes and 53 common analyzed CDS (GC_g_ and GC_c_), as well as GC contents of the first, second, and third codon positions of analyzed CDS (GC_1_, GC_2_ and GC_3_, respectively) were calculated by GENEIOUS R8. Relative synonymous codon usage (RSCU) is the ratio of the observed frequency of a codon to the expected frequency and is a good indicator of codon usage bias (Sharp and Li, 1986). When synonymous codons are used less frequently than expected, RSCU value is less than 1, otherwise the value is greater than 1 (Gupta et al., 2004). The above work was completed by MEGA 5.0 (Tamura et al., 2011).

### Repeat elements analysis

REPuter (Kurtz et al., 2001) was used to identify dispersed and palindromic repeats within plastid genomes. We focused on the repeats having a minimal size of 30 bp and 90% or greater similarity between the two repeat copies. The maximum distance between palindromic repeats is 3 Kb. Tandem repeats (>10 bp in length) were detected using online program Tandem Repeats Finder (TRF) (Benson, 1999) with default parameters. The minimum alignment score and maximum period size set as 80 and 500, respectively. All of the above parameters were set based on some related plastid studies (Huang et al., 2013, 2014; Rousseau-Gueutin et al., 2015). All found repeats were manually verified and the redundant results were removed. In Yang et al. (2016), all three types of repeats had been identified in *Q. dolicholepis, Q. variabilis, Q. aliena, Q. aliena* var. *acuteserrata* and *Q. baronii*, which were processed in the same way as this study. Therefore, repeat elements were detected only in the other 11 plastid genomes.

### Sequence divergence analysis

Sequence divergence was evaluated for protein-coding sequences by calculating pairwise distance between each two species. Pairwise distances were calculated using MEGA 5.0 with K2p evolution model (Kimura, 1980). A visual alignment of complete plastid genomes was generated in mVISTA (Frazer et al., 2004).

### Phylogenetic analysis

To evaluate the effect of codon composition bias and gene function on phylogenetic estimation, we respectively constructed the aligned matrices of shared protein-coding genes, codon positions 1 + 2, codon position 3, and 5 functional categories of protein-coding genes (Chang et al., 2006; Liu et al., 2012) for the Fagaceae phylogeny. All of the above analyzed matrices were obtained from 76 shared protein-coding genes. The extraction of different positions of codon was conducted by MEGA 5.0. *Populus trichocarpa* (EF489041) (Tuskan et al., 2006) and *Theobroma cacao* (HQ244500) were chosen as outgroups. Sequence alignment was performed using MAFFT (Katoh and Standley, 2013) in GENEIOUS R8 with the default parameters set.

All phylogenetic analyses were performed using maximum likelihood (ML) methods and Bayesian inference (BI), which were conducted using RAxML v7.2.8 (Stamatakis, 2006) and MrBayes v3.1.2 (Ronquist and Huelsenbeck, 2003), respectively. The ML tree was inferred with GTR+G model and 1000 rapid bootstrap replicates. The best-fitting model for BI analyses was determined using Modeltest 3.7 (Posada and Crandall, 1998) based on the Akaike information criterion (AIC). Two independent Markov chain Monte Carlo (MCMC) runs were performed for 2 million generations with sampling every 100 generations, and the first 25% of the trees were discarded as burn-in.

## Results

### Plastid assembly, genome characteristics, and codon usage bias

Four plastids (*F. engleriana, Q. spinosa, Q. aquifolioides*, and *Q. glauca*) were generated in the current study. Illumina sequencing produced large data sets. 362,613 (*F. engleriana*) to 788,550 (*Q. aquifolioides*) reads were assembled to generate the plastid genomes, ranging from 281 × to 616 × coverage (Table 1). These plastid genomes possess the typical quadripartite structure, ranging from 158,346 bp (*F. engleriana*) to 161,225 bp (*Q. aquifolioides*) (Table 2). Except *F. engleriana*, the other three plastid genomes share identical gene content and gene order, encoding a total of 134 genes, including 86 protein-coding genes (CDS), 40 transfer RNA (tRNA) genes, and 8 ribosomal RNA (rRNA) genes (Table 2). *Fagus engleriana* encodes a total of 131 genes, containing the same numbers of tRNA and rRNA genes except for three lost protein-coding genes (*rps16, infA*, and *rpl22*) compared with the other three species.

**Table 2:** Characteristics of Fagaceae plastid genomes.

**Species**	**Genome size (bp)**	**LSC (bp)**	**SSC (bp)**	**IR (bp)**	**Number of genes**	**Pseudo gene**	**Number of protein-coding genes**	**Number of tRNA genes**	**Number of rRNA genes**
*Quercus rubra*	161,304	90,541	19,025	51,738	137	/	89	40	8
*Quercus aliena*	161,150	90,444	19,054	51,652	134	/	86	40	8
*Quercus aliena* var. *acuteserrata*	161,153	90,457	19,044	51,652	134	/	86	40	8
*Quercus baronii*	161,072	90,341	19,045	51,686	134	/	86	40	8
*Quercus dolicholepis*	161,237	90,461	19,048	51,728	134	/	86	40	8
*Quercus variabilis*	161,077	90,387	19,056	51,634	134	/	86	40	8
Quercus aquifolioides^*^	161,225	90,535	19,000	51,690	134	/	86	40	8
Quercus spinose^*^	161,156	90,441	18,997	51,718	134	/	86	40	8
Quercus glauca^*^	160,798	90,229	18,907	51,662	134	/	86	40	8
*Quercus edithiae*	160,988	90,352	18,954	51,682	128	*rpl22, ycf15*(x2)	87	30	8
*Castanea mollissima*	160,799	90,432	18,995	51,372	130	*rpl22, ycf1*	83	37	8
*Castanea pumila* var. *pumila*	160,603	90,249	18,976	51,378	131	*rpl22*	83	39	8
*Castanopsis echinocarpa*	160,647	90,394	18,995	51,258	132	/	84	40	8
*Lithocarpus balansae*	161,020	90,596	19,160	51,264	134	/	87	39	8
*Trigonobalanus doichangensis*	159,938	89,374	19,292	51,272	128	/	81	39	8
Fagus engleriana^*^	158,346	87,667	18,895	51,784	131	/	83	40	8

The 4 newly generated plastid genomes were marked in ^*^.

**Figure 1:**
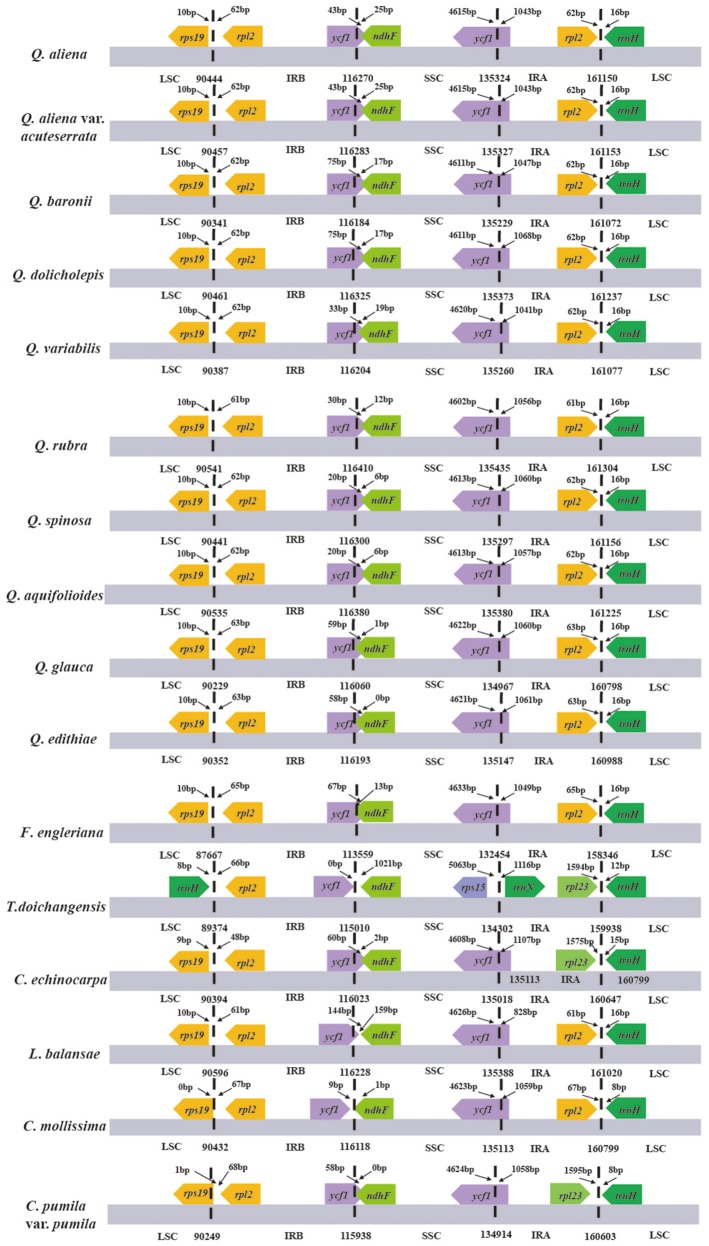
The comparison of the LSC, IR, and SSC border regions among the Fagaceae plastid genomes. Numbers above the gene features mean the distance from the end of gene to the boundary region. These features are not to scale.

A comparison of the major characteristics of all available Fagaceae plastid genomes is shown in Table 2. *F. engleriana* has the smallest plastid genome (158,346 bp), whereas *Q. rubra* has the largest (161,304 bp). The number of encoded genes varies from 128 (*T. doichangensis*) to 137 (*Q. rubra*). In particular, the number of tRNA genes in *Q. edithiae* is significantly decreased compared with other species. Gene differences are provided as Supplemental Data (Table S1). There exist pseudogenes in *Q. edithiae, C. mollissima* and *C. pumila* var. *pumila*. The IR/SC boundary regions in Fagaceae show slight differences (Figure 1). For example, the extended length of *ycf1* into SSC region range from 0 (*T. doichangensis*) to 144 bp (*L. balansae*).

Overall, GC content levels of different species are very close in the same region (such as CDS, different codon positions; Table 3). Both the genome-wide GC content (GC_g_) (about 36.8%) and CDS GC content (GC_c_) (about 38.6%) indicate that the plastid genome is AT-rich. Within the analyzed CDS, the mean values of GC content for the first, second and third codon positions of 16 Fagaceae species are 46.4, 38.4, and 31.0%, respectively.

**Table 3:** GC content of sequences in Fagaceae plastid genomes.

**Species**	**Number of analyzed CDS**	**GCg (%)**	**GCc (%)**	**GC_1_ (%)**	**GC_2_ (%)**	**GC_3_ (%)**
*Quercus rubra*	53	36.8	38.6	46.3	38.3	31.0
*Quercus aliena*	53	36.8	38.6	46.4	38.4	30.9
*Quercus aliena* var. *acuteserrata*	53	36.8	38.6	46.4	38.4	30.9
*Quercus baronii*	53	36.8	38.6	46.4	38.4	31.0
*Quercus dolicholepis*	53	36.8	38.6	46.4	38.4	30.9
*Quercus variabilis*	53	36.8	38.6	46.4	38.4	31.0
*Quercus aquifolioides*	53	36.8	38.6	46.4	38.4	31.0
*Quercus spinosa*	53	36.8	38.6	46.4	38.3	31.0
*Quercus glauca*	53	36.9	38.6	46.5	38.4	31.0
*Quercus edithiae*	53	36.9	38.6	46.5	38.4	31.0
*Castanea mollissima*	53	36.8	38.5	46.4	38.3	30.9
*Castanea pumila* var. *pumila*	53	36.8	38.5	46.3	38.3	30.9
*Castanopsis echinocarpa*	53	36.7	38.6	46.4	38.4	31.0
*Lithocarpus balansae*	53	36.7	38.5	46.3	38.3	30.9
*Trigonobalanus doichangensis*	53	37.0	38.8	46.6	38.5	31.3
*Fagus engleriana*	53	37.1	38.5	46.5	38.3	30.8

*GCg: GC content of whole genome; GC_c_: GC content of 53 analyzed CDS; GC_1_, GC_2_, GC_3_: GC content at first, second and third codon positions, respectively*.

For the analyzed CDS, the frequency of codon usage in each species is summarized in Table S2. Codon usage bias is fairly similar across Fagaceae. In all species, the most and least prevalent amino acids always are leucine (approximately 10.5%) and cysteine (approximately 1.2%), respectively. Moreover, except Met and Trp that are encoded by only one codon, all the other amino acids show that some codons appear to be used more frequently than others. For example, synonymous codons UUA, UUG, CUU, CUC, CUA and CUG encode leucine and the corresponding RSCU values for these six codons in *F. engleriana* are 1.83, 1.24, 1.31, 0.40, 0.82, and 0.40, respectively, as expected from the low GC content of CDS.

### Repeat elements

A total of 440 repeat elements are identified for these three repeat types in the 16 complete plastid genomes (Table 4). The numbers of tandem, dispersed, and palindromic repeats are 145, 199 and 96, respectively. IR regions have the most repeats (220, 50.0%), followed with LSC (170, 38.6%) and SSC (50, 11.4%). From another point of view, the majority of repeats are located in intergenic spacer regions (234, 53.2%), and the minority are found in introns (89, 20.2%). Ratios of number of repeat bases to number of bases in the region (number of repeat bases / number of bases in the region) for different region comparisons show that IR regions and introns host the highest ratios (1.66 and 1.88%, respectively). Only a few genes (e.g., *ycf1, ycf2*, atpF, *psaA, psaB*, and some tRNA genes) possess repeat elements. All dispersed and palindromic repeats occur in a narrower size (30–40 bp), except for a 72 bp dispersed repeat in *Q. edithiae*. Regarding the tandem repeats, shorter repeats are common (< 40 bp), whereas only 5 longer repeats (> 40 bp) are detected (4 in *Q. edithiae* and 1 in *F. engleriana*). Moreover, the majority of 10–20 bp tandem repeats and 21–30 bp tandem repeats are found in introns and genes, respectively. Overall, number and distribution of repeat elements are conserved across these Fagaceae species (Table S3).

**Table 4:** Analyses of repeat elements in Fagaceae plastid genomes.

**Location**	**Tandem repeats**		**Dispersed repeats**		**Palindromic repeats**		**All kinds of repeats**
	**Number of different length repeats (10–20 bp/21–30 bp/31–40 bp/>40 bp)**	**Number of repeat bases/number of bases in the region**	**Number of different length repeats (30–40 bp/>40 bp)**	**Number of repeat bases/number of bases in the region**	**Number of different length repeats (30–40 bp/>40 bp)**	**Number of repeat bases/number of bases in the region**	**Number of repeat bases/number of bases in the region**
Complete plastid genomes	145 (53/50/37/5)	8,459/2,572,513	199 (198/1)	12,832/2,572,513	96 (96/0)	6,150/2,572,513	27,441/2,572,513
LSC	40 (20/12/4/4)	2219/1,442,900	97 (97/0)	6,081/1,442,900	33 (33/0)	2,050/1,442,900	10,350/1,442,900
SSC	11 (3/6/1/1)	634/304,443	21 (20/1)	1,670/304,443	18 (18/0)	1,090/304,443	3,394/304,443
IR	94 (30/32/32/0)	5,606/825,170	81 (81/0)	5,081/825,170	45 (45/0)	3,010/825,170	13,697/825,170
Intergenic spacer regions	79 (25/17/33/4)	4,451/828,186	95 (95/0)	6,289/828,186	60 (60/0)	4,094/828,186	14,834/828,186
Introns	28 (26/0/1/1)	1,388/284,012	41 (40/1)	2,849/284,012	20 (20/0)	1,096/284,012	5,333/284,012
Genes	38 (2/33/3/0)	2,620/1,460,315	63 (63/0)	3,694/1,460,315	16 (16/0)	960/1,460,315	6,714/1,460,315

*Numbers of different length repeats are given in brackets*.

### Sequence divergence

**Figure 2:**
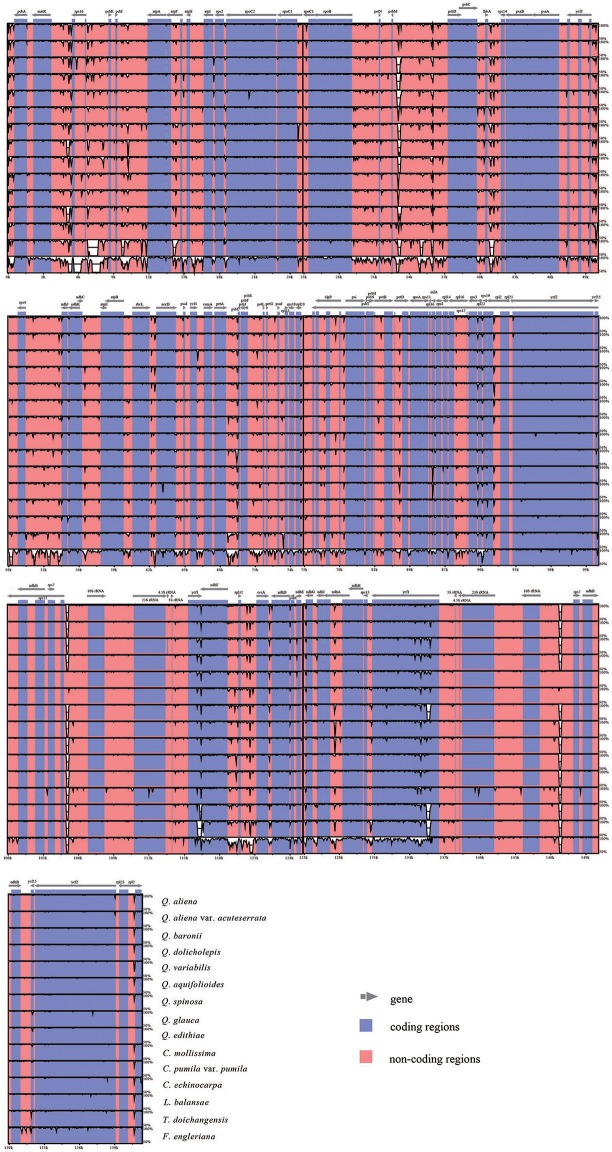
Sequence identity plot comparing the 16 Fagaceae plastid genomes with *Q. rubra* as a reference. The y-axis represents % identity ranging from 50 to 100%. Coding and noncoding regions are marked in purple and pink, respectively.

With *Q. rubra* as a reference, the alignment of 16 complete plastid genomes is performed using mVISTA (Figure 2). Overall, sequence divergence is low across the Fagaceae plastid genomes. Among them, *F. engleriana* shows marked differences compared with other species. As expected, IRs and coding regions exhibit higher conservation than SC regions and noncoding regions, respectively. For the conservation of IR regions, the substitution rates in SC regions have been detected to be several times higher than that in IR regions among diverse plants (Zhu et al., 2015), and a copy-dependent repair mechanism has been proposed to explain the lower substitution rate in IR (Perry and Wolfe, 2002). Pairwise comparisons of genetic divergence are estimated by K2p distance, ranging from 0 (*Q. aliena* vs. *Q. aliena* var. *acuteserrata*) to 0.032 (*F. engleriana* vs. *T. doichangensis*) (Table S4). In general, low genetic divergence occurs in Fagaceae. However, when *F. engleriana* is included, the values of genetic divergence are always high (vary from 0.029 to 0.032). *T. doichangensis* is another taxon that shows relatively high genetic divergence (approximately 0.007). Interestingly, the infrageneric divergence in *Quercus* (ranges from 0.001 to 0.005) is comparable to that of inter-generic differentiation in Fagaceae (e.g., distance between *Lithocarpus* and *Castanopsis* is 0.004, distance between *Castanopsis* and *Castanea* is 0.003).

### Fagaceae phylogeny

Different analysis methods (BI and ML analyses) yield largely identical phylogenetic trees from each dataset [76 shared protein-coding genes, codon positions 1 + 2, codon position 3, and five functional categories of protein-coding genes (Table 5)]. The aligned length and used model of each dataset are shown in Table 6. The aligned sequences of the first three datasets are shown in Supplemental Data Sheet 1.

**Table 5:** List of the 76 common protein-coding genes divided into five functional groups.

**Protein-coding gene category**	**Genes**
Gene expression	*rps2, rps14, rps4, rps18, rps12, rps11, rps8, rps3, rps19, rps7, rps15, rps7, rpl33, rpl20, rpl36, rpl14, rpl16, rpl2, rpl23* (^*^2), *rpoC2, rpoC1, rpoB, rpoA*
Photosynthetic apparatus	*psbA, psbK, psbI, psbM, psbD, psbC, psbJ, psbL, psbF, psbE, psbB, psbT, psbN, psbH, psaB, psaA, psaI, psaJ, psaC, petN, petA, petL, petB, petD, ycf3, ycf4, accD*
Photosynthetic metabolism	*atpA, atpF, atpH, atpI, atpE, atpB, rbcL, ndhJ, ndhK, ndhC, ndhB* (^*^2), *ndhF, ndhD, ndhE, ndhG, ndhI, ndhA, ndhH*
Miscellaneous	*matK, cemA, clpP, ccsA*
Unknown	*ycf2* (^*^2)

*Numbers in parentheses indicate the genes duplicated in the IR regions*.

**Table 6:** Sites and models in ML and BI analyses for each dataset.

**Dataset**	**Number of sites**	**Model in ML**	**Model in BI**
76 common protein-coding genes	72,235	GTR+G	GTR+I+G
Codon positions 1 + 2	48,176	GTR+G	TVM+I+G
Codon position 3	24,353	GTR+G	GTR+G
Gene expression	18,856	GTR+G	GTR+I+G
Photosynthetic apparatus	16,970	GTR+G	GTR+G
Photosynthetic metabolism	18,817	GTR+G	TVM+I+G
Miscellaneous	3,786	GTR+G	TVM+G
Unknown	13,914	GTR+G	GTR

**Figure 3:**
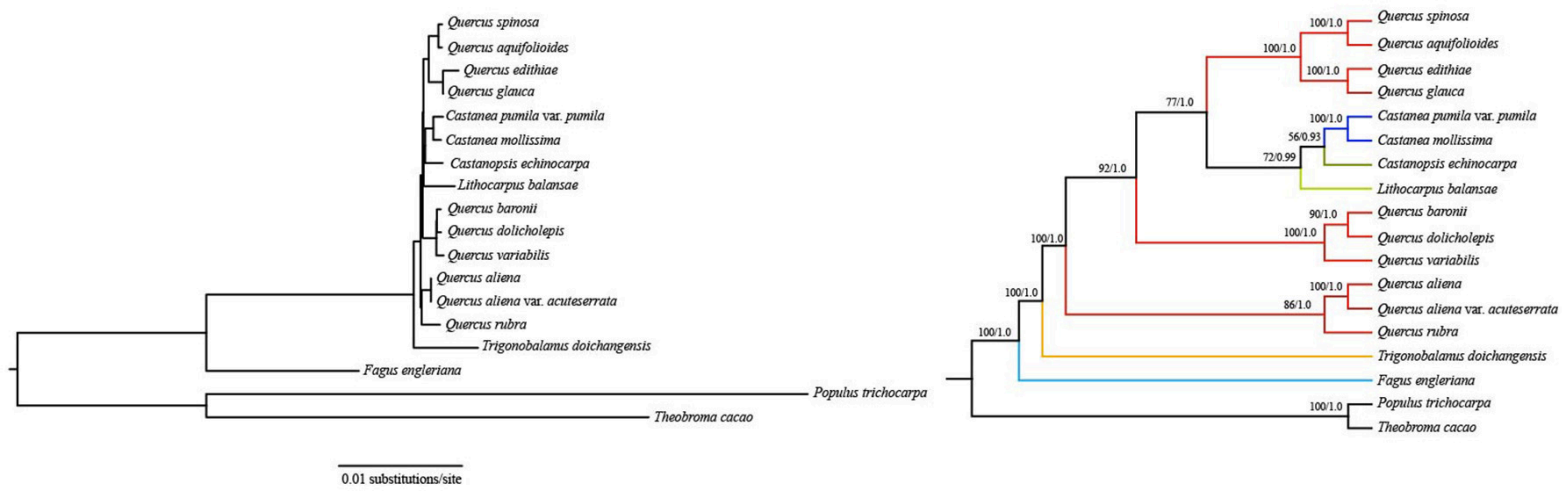
Fagaceae phylogeny based on ML and BI analyses of 76 protein-coding genes. ML topology shown with bootstrap support values and posterior probability values listed at each node.

Support is generally high for almost all relationships inferred from 76 common protein-coding genes (the support values have a range of 72/0.99–100/1.0, except for a node with 56/0.93 support) (Figure 3). *F. engleriana* is in the basal position, followed by *T. doichangensis*. *Lithocarpus balansae* is sister to a clade of (*Castanopsis echinocarpa, C. mollissima, Castanea pumila* var. *pumila*). It is noteworthy that species in the genus *Quercus* do not form a clade. The 3rd codon site dataset and five functional groups of protein-coding genes datasets exhibit partly congruent versions compared with the above topology (Figures S1–S6). Differences mainly include the positions of groups in *Quercus* and the corresponding nodes obtain weak-to-moderate support (support values are generally < 50/0.50). Moreover, the topologies of species in a group (such as in *Quercus*) or in a genus (such as *Castanea*) are identical in almost all analyses and receive strong support.

**Figure 4:**
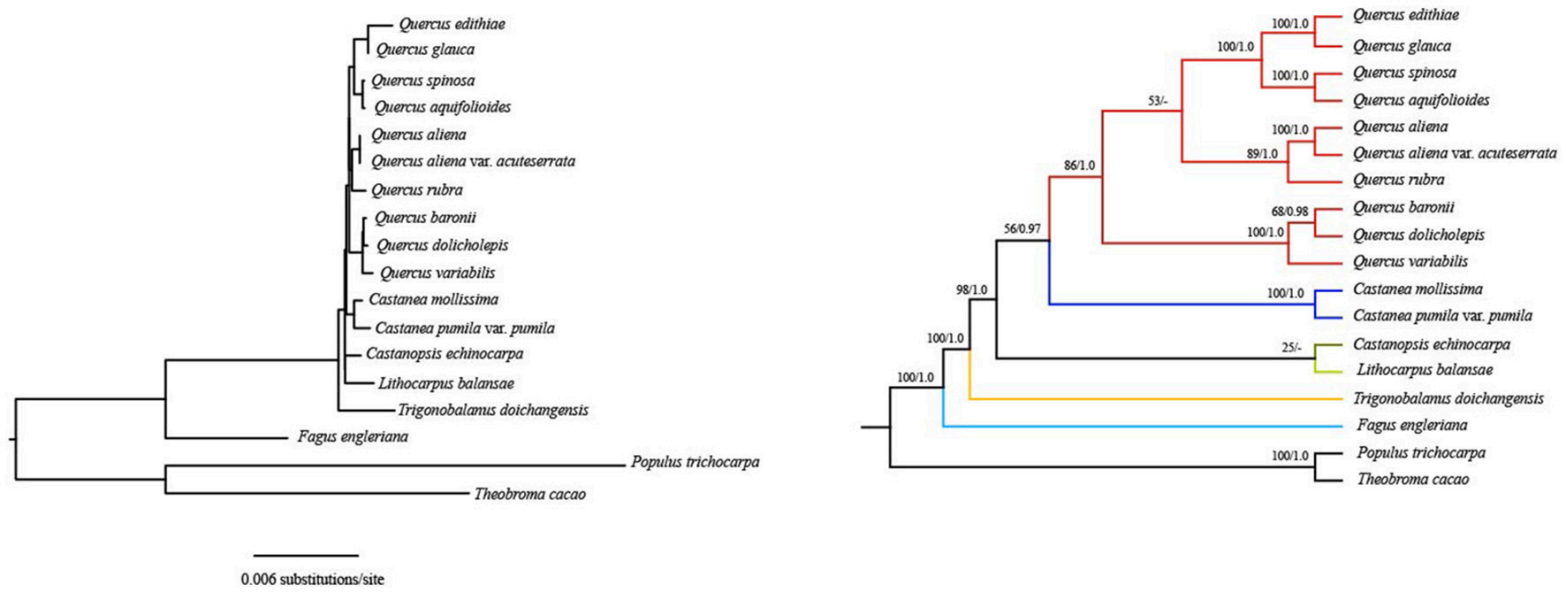
Fagaceae phylogeny based on ML and BI analyses of the first two codon positions of protein-coding genes. ML topology shown with bootstrap support values and posterior probability values listed at each node. Dash denotes nodes contradicted by the BI trees with posterior probability values < 0.50.

Notably, phylogenetic relationships derived from the first two codon sites dataset are completely recovered with generally strong support and all oaks form a clade with high support (86% bootstrap values and 1.0 posterior probabilities) (Figure 4). These *Quercus* species are divided into two clades. The first clade split into two subclades: one shows that *Q. rubra* is sister to *Q. aliena* and *Q. aliena* var*. acuteserrata*; the other shows that *Q. baronii* appears to be more closely related to *Q. dolicholepis* than to *Q. variabilis*. The second clade is composed of group *Cyclobalanopsis* (according to Denk and Grimm, 2010) (*Q. glauca* and *Q. edithiae*) and species *Q. spinosa* and *Q. aquifolioides*. Overall, the topology of other clades (genus *Fagus, Trigonobalanus, Lithocarpus*, and *Castanopsis*) is nearly identical to those based on two nuclear loci (ITS and *CRC*) (Oh and Manos, 2008), except for the placement of *Castanea* as sister to *Quercus* vs. *Castanopsis*.

## Discussion

### Plastid sequence evolution

In general, the size, gene content and gene order are similar among the plastid genomes, which reveal that plastid genomes are highly conserved in Fagaceae. Moreover, gene loss occurs in Fagaceae (Table S1). From the result of alignment, we find that the lost protein-coding genes are caused by annotation error in most cases (e.g., the lost protein-coding genes *ycf1, rpl2, rpl22, petG*). Firstly, the sequences that encode the lost genes not only possess proper initial and termination codons, but also present highly conserved content compared with other species. Furthermore, the protein-coding gene loss only occurs in one or two species, whereas the corresponding protein-coding genes always exist in the other species.

IR contraction and expansion is a common evolutionary phenomenon (Kim and Lee, 2004; Hansen et al., 2007; Wang et al., 2008; Davis and Soreng, 2010; Huang et al., 2014) and may cause variation in length of angiosperm plastid genome (Kim and Lee, 2004). The slight differences of IR/SC boundary regions in Fagaceae may be the result of IR contraction/expansion. Moreover, the minor IR boundary shifts of Fagaceae plastomes have neither triggered the transfer of genes between SC regions and IR regions or the gain/loss of genes, which have been detected in some plant lineages (Zhu et al., 2015, and references therein).

Codon usage bias is an important evolutionary phenomenon. GC content is the major factor in shaping the biased codon usage and could play an important role during the evolution of genomic structure (e.g., thermostability and modulation of replication, transcription and translation) (Sueoka and Kawanishi, 2000; Bellgard et al., 2001, and references therein). The observation of GC content level indicates that plastid genome in Fagaceae are AT-rich and there is a strong bias toward A/T at the third codon position, which are consistent with previous plastid genome studies (e.g., Shimada and Sugiuro, 1991; Clegg et al., 1994; Tangphatsornruang et al., 2009; Delannoy et al., 2011). The presence of translation-preferred codons may be the result of both natural selection and mutation preference during the plastid genome evolutionary process. Variations in codon bias are highly similar in all analyzed species, which also suggests that Fagaceae plastid genomes are highly conserved.

Larger and more complex repeat sequences may play an important role in the rearrangement of plastid genomes and sequence divergence (Timme et al., 2007; Weng et al., 2013); therefore, we investigated the numbers and distributions of tandem, dispersed, and palindromic repeats. We find that repeats in different species are usually located in the same genes (*ycf1* and *ycf2*), or genes with similar functions (e.g., *psaB*/*psaA, trnS*-*GCU/trnS*-*UGA, trnG-GCC/trnG-UCC*, and *trnS-UGA/trnS-GGA*). Moreover, longer repeats are rare in Fagaceae plastomes (6 of the 440 repeats are longer than 40 bp) compared with some other plant lineages (Zhang et al., 2011; Huang et al., 2014; Cai et al., 2015)

Overall, low genetic divergence occurs in Fagaceae. *Fagus* represents an early diverged group in Fagaceae (Manos et al., 2001), which may result in relatively high genetic divergence between *F. engleriana* and other species. The infrageneric divergence in *Quercus* is comparable to that of inter-generic differentiation in Fagaceae, which was also observed in the studies of Simeone et al. (2016) and Vitelli et al. (2017). As a widely distributed genus, the relatively high inter-specific variation in *Quercus* may be related to the local adaptation to different environments. Recently, adaptive genetic variation of several climate-associated genes in oaks have been detected (Sork et al., 2015; Rellstab et al., 2016).

### Fagaceae phylogeny and the effects of codon composition bias and gene function

The phylogenetic tree based on the 76 shared protein-coding genes receives generally strong support. The closer relationships among genera *Lithocarpus, Castanopsis*, and *Castanea* in this study support the taxonomic treatment of insect-pollinated subfamily Castaneoideae, including *Chrysolepis, Lithocarpus, Castanopsis*, and *Castanea* (Nixon, 1989; Oh and Manos, 2008). Notably, genus *Quercus* has always been resolved as monophyletic in the previous nuclear phylogenies (Oh and Manos, 2008; Denk and Grimm, 2010; Hubert et al., 2014), however, infragenetic groups of *Quercus* do not form one clade in this study (Figure 3). This phenomenon was also observed in previous molecular phylogenies (e.g., Manos et al., 2008; Simeone et al., 2016). In sum, resemblance between nuclear gene tree and plastid tree of genus *Quercus* is lost. Beside the possible reasons mentioned in the introduction (e.g., chloroplast capture, incomplete lineage sorting, and different evolutionary histories of plastid and nucleus), the complex evolutionary history of oaks (Jiménez et al., 2004; Grivet et al., 2006) may also be taken into account.

While the 76 common protein-coding genes dataset generates a highly supported phylogeny, the inference may be an artifact when considering the topology of the genus *Quercus* as inferred from nuclear genes and pollen morphology (Oh and Manos, 2008; Denk and Grimm, 2009, 2010). Thus, we further evaluated the impact of codon composition bias and gene function, which may have influence on topological structure. Phylogenetic trees derived from the third codon position and five functional categories of protein-coding genes not only fail to resolve all oaks as one clade, but also show conflicting relationships in some clades (with weak-to-moderate support). For the third codon position, so much change has occurred at these sites as they are near neutral (Sueoka, 1988). Thus, the biased inference may be attributed to less historically accurate information provided by these sites (Cox et al., 2014). From the results of the phylogenetic trees based on different gene function datasets, we concluded that a relatively small number of plastid genes did not provide sufficient phylogenetic signal to explore the relationships in this complex and long-lived woody plants. In other words, gene function is not the determining factor that influences Fagaceae phylogenetic inference. Moreover, we also used RY recoding (A and G = R, C and T = Y) to analyze the 76 shared protein-coding genes. However, the recovered tree was not better than the trees obtained on the original dataset or with codon positions 1+2. In particular, the genus Quercus was not monophyletic (data not shown).

Using the first two codon sites dataset, relationships are completely recovered with generally strong support and all oaks form one clade, which is compatible with the more plausible nuclear phylogeny (Oh and Manos, 2008). The first and second codon positions are subject to functional constraints against non-synonymous mutation, because mutations at these positions usually lead to amino acid change. For many phylogenetic analyses, it is common to eliminate the third codon position considering the effect of composition bias (Goremykin et al., 2003; Gibson et al., 2005; Cox et al., 2014). In the phylogenetic tree generated from the dataset considering only the first and second codon sites, *F. engleriana* is the first to diverge, followed by *T. doichangensis*, which indicates that they are early-diverging taxa in Fagaceae. This is in agreement with the recent discovery of the oldest known *Fagus* remains from ca. 60 Ma old sedimentary rocks of western Greenland (Grímsson et al., 2016). Although the phylogenetic tree yields a sister relationship between genus *Castanea* and genus *Quercus*, the support values of the node are poor (56% bootstrap value). Thus, we do not conclude that *Castanea* appears to be more closely related to *Quercus* than to *Castanopsis*. Overall, all of the relationships among these genera are nearly identical to those inferred from nuclear data (Oh and Manos, 2008; Denk and Grimm, 2010). In the genus *Quercus*, based on pollen characteristics and nuclear markers, six major intrageneric groups (*Cyclobalanopsis, Cerris, Ilex, Lobatae, Protobalanus*, and *Quercus*) have been identified (Oh and Manos, 2008; Denk and Grimm, 2009, 2010; Hubert et al., 2014). Relationships among *Q. rubra, Q. aliena, Q. aliena* var. *acuteserrata, Q. baronii, Q. dolicholepis*, and *Q. variabilis* in the current study are identical to that in Yang et al. (2016), which were inferred from complete plastid genome sequences and different plastid genome regions (LSC+SSC+IRB, LSC+SSC, LSC, SSC). However, the positions of *Q. spinosa* and *Q. aquifolioides* were either unresolved or poorly supported in Yang et al. (2016). Herein, the two species always form a well-supported monophyletic clade and then cluster with group *Cyclobalanopsis*. *Q. baronii, Q. dolicholepis, Q. spinosa* and *Q. aquifolioides* are regarded as members of group *Ilex* in earlier studies (Denk and Grimm, 2009, 2010; Simeone et al., 2013; Denk and Tekleva, 2014; Hubert et al., 2014), while they do not cluster together in this phylogenetic tree. *Q. baronii* and *Q. dolicholepis* appear more closely related to *Q. variabilis*, which belongs to group *Cerris*. Based on nuclear genes or plastid markers, Asian species (e.g., *Q. pseudosemicarpifolia, Q. semecarpifolia, Q. franchetii* sampled from China) in group *Ilex* were always embedded in group *Cerris* (Simeone et al., 2013, 2016; Hubert et al., 2014). It is possible that incomplete lineage sorting and introgression cause this scenario. In another cluster, *Q. rubra* shows closer relationship to *Q. aliena* and *Q. aliena* var*. acuteserrata* (sampled from China). Group *Lobatae* occurs in New World only and group *Quercus* occurs both in the Old and New World; the ancestral area of these two groups is North America with dispersal to Asia and then Europe, which may contribute to the widespread distribution of group *Quercus* (Manos and Stanford, 2001). Previous molecular studies demonstrated that there was generally low genetic differentiation between North American and Eurasian members of group *Quercus* (Manos et al., 2001; Denk and Grimm, 2010). There were generally closer relationships among New World groups (*Lobatae, Protobalanus*, and *Quercus*) based on pollen characteristics and molecular markers, as we found in our study. Certainly, it would be necessary to sample more species to explore the phylogenetic relationships of *Quercus* in future.

## Author contributions

YY and GZ: designed the experiments; YY, JZ, LF, TZ, GB, and JY: performed the experiments and analyzed the data; YY: wrote the paper; All authors read and approved the final manuscript.

### Conflict of interest statement

The authors declare that the research was conducted in the absence of any commercial or financial relationships that could be construed as a potential conflict of interest.

## Supplementary material

Figure S1 Fagaceae phylogeny based on ML and BI analyses of the third codon position of protein-coding genes. ML topology shown with bootstrap support values and posterior probability values listed at each node. Dash denotes nodes contradicted by the BI trees with posterior probability values < 0.50.

Figure S2 Fagaceae phylogeny based on ML and BI analyses of genes belonging to gene expression functional category. ML topology shown with bootstrap support values and posterior probability values listed at each node. Dash denotes nodes contradicted by the BI trees with posterior probability values < 0.50.

Figure S3 Fagaceae phylogeny based on ML and BI analyses of genes belonging to photosynthetic apparatus functional category. ML topology shown with bootstrap support values and posterior probability values listed at each node. Dash denotes nodes contradicted by the BI trees with posterior probability values < 0.50.

Figure S4 Fagaceae phylogeny based on ML and BI analyses of genes belonging to photosynthetic metabolism functional category. ML topology shown with bootstrap support values and posterior probability values listed at each node. Dash denotes nodes contradicted by the BI trees with posterior probability values < 0.50.

Figure S5 Fagaceae phylogeny based on ML and BI analyses of genes belonging to miscellaneous functional category. ML topology shown with bootstrap support values and posterior probability values listed at each node. Dash denotes nodes contradicted by the BI trees with posterior probability values < 0.50.

Figure S6 Fagaceae phylogeny based on ML and BI analyses of genes belonging to unknown functional category. ML topology shown with bootstrap support values and posterior probability values listed at each node. Dash denotes nodes contradicted by the BI trees with posterior probability values < 0.50.

Table S1 Gene differences compared with *Quercus baronii*.

Table S2 Summary of average relative synonymous codon usage (RSCU) of the codon usage in the Fagaceae cp genomes. AA, Amino acid; Fe, *F. engleriana*; Td, *T. doichangensis*; Qr, *Q. rubra*; Qs, *Q. spinosa*; Qa, *Q. aquifolioides*; Qb, *Q. baronii*; Qal, *Q. aliena*; Qala, *Q. aliena* var. *acuteserrata*; Qv, *Q. variabilis*; Qd, *Q. dolicholepis*; Qg, *Q. glauca*; Qe, *Q. edithiae*; Ce, *C. echinocarpa*; Lb, *L. balansae*; Cm, *C. mollissima*; Cp, *C. pumila* var. *pumila*.

Table S3 Repeats distribution.

Table S4 Protein-coding sequence divergence (K2p) in Fagaceae.
