# Supplementary figures and images for: Plastid Genome Comparative and Phylogenetic Analyses of the Key Genera in Fagaceae: Highlighting the Effect of Codon Composition Bias in Phylogenetic Inference
Source: Front Plant Sci. 2018 Feb 1;9:82. doi: 10.3389/fpls.2018.00082 (PMC5800003; doi:10.3389/fpls.2018.00082)

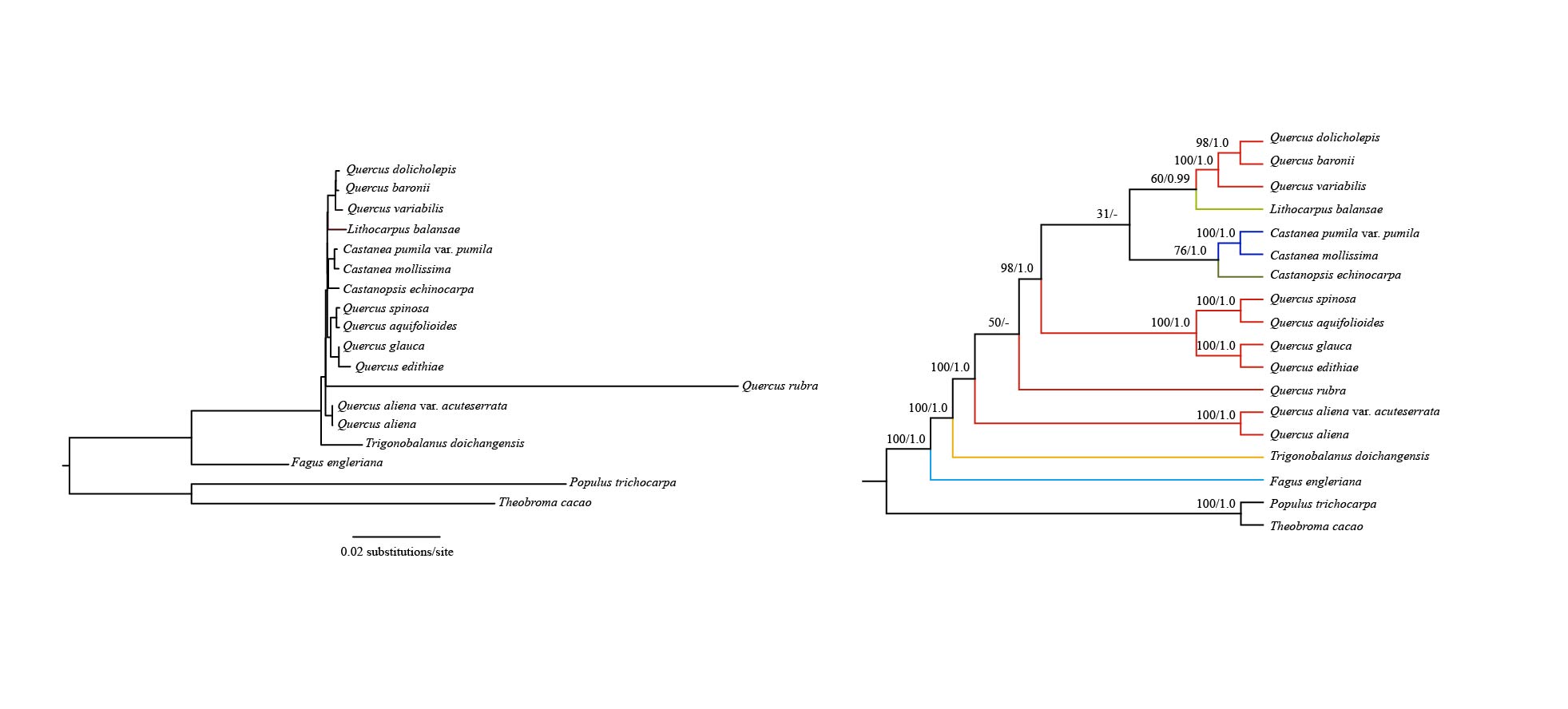

Supplement: Figure S1 — Fagaceae phylogeny based on ML and BI analyses of the third codon position of protein-coding genes. ML topology shown with bootstrap support values and posterior probability values listed at each node. Dash denotes nodes contradicted by the BI trees with posterior probability values < 0.50. [file Image1.JPEG]

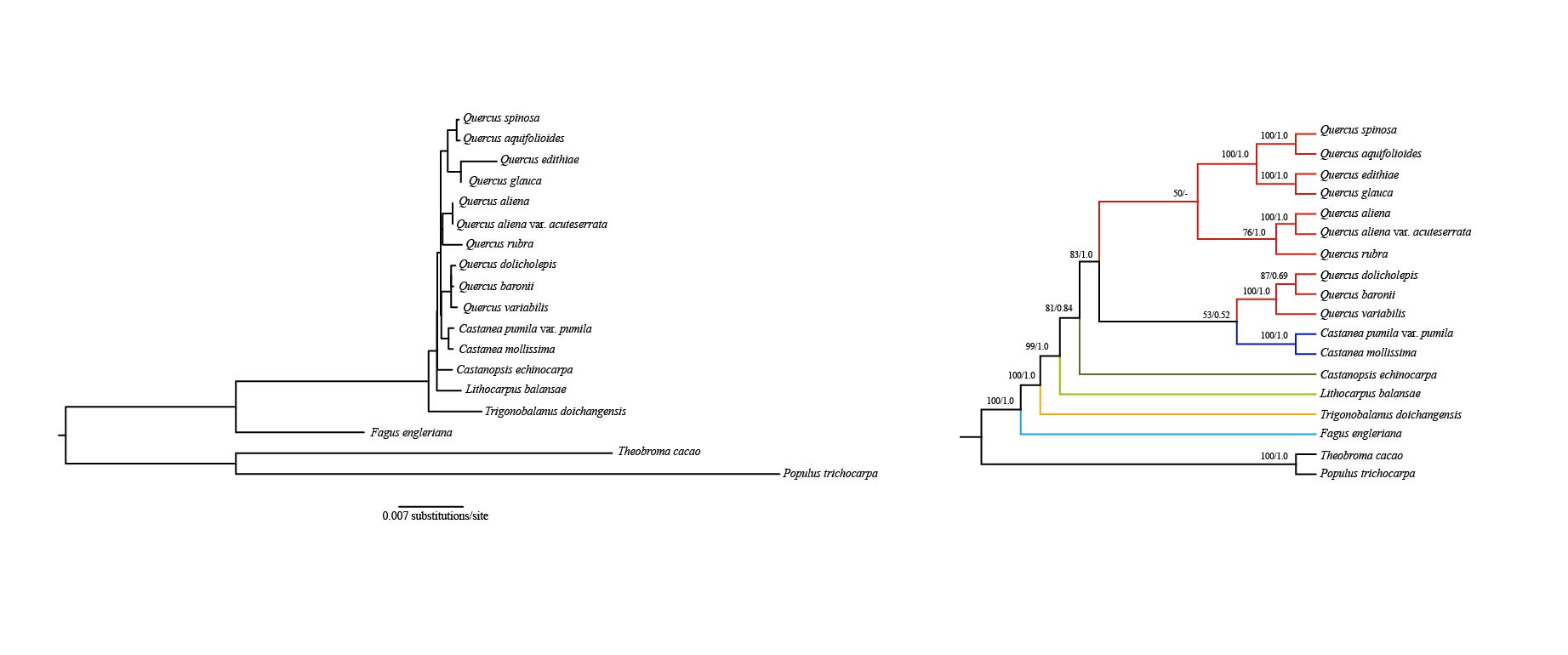

Supplement: Figure S2 — Fagaceae phylogeny based on ML and BI analyses of genes belonging to gene expression functional category. ML topology shown with bootstrap support values and posterior probability values listed at each node. Dash denotes nodes contradicted by the BI trees with posterior probability values < 0.50. [file Image2.JPEG]

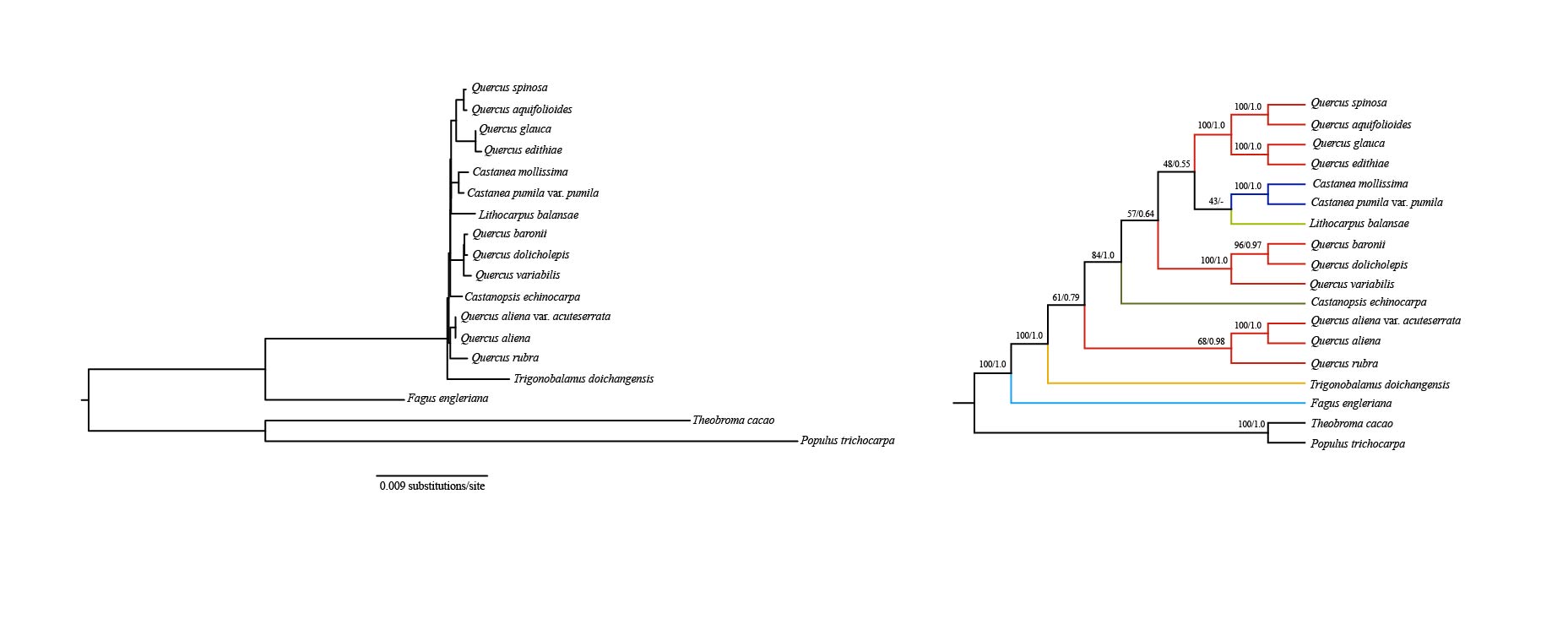

Supplement: Figure S3 — Fagaceae phylogeny based on ML and BI analyses of genes belonging to photosynthetic apparatus functional category. ML topology shown with bootstrap support values and posterior probability values listed at each node. Dash denotes nodes contradicted by the BI trees with posterior probability values < 0.50. [file Image3.JPEG]

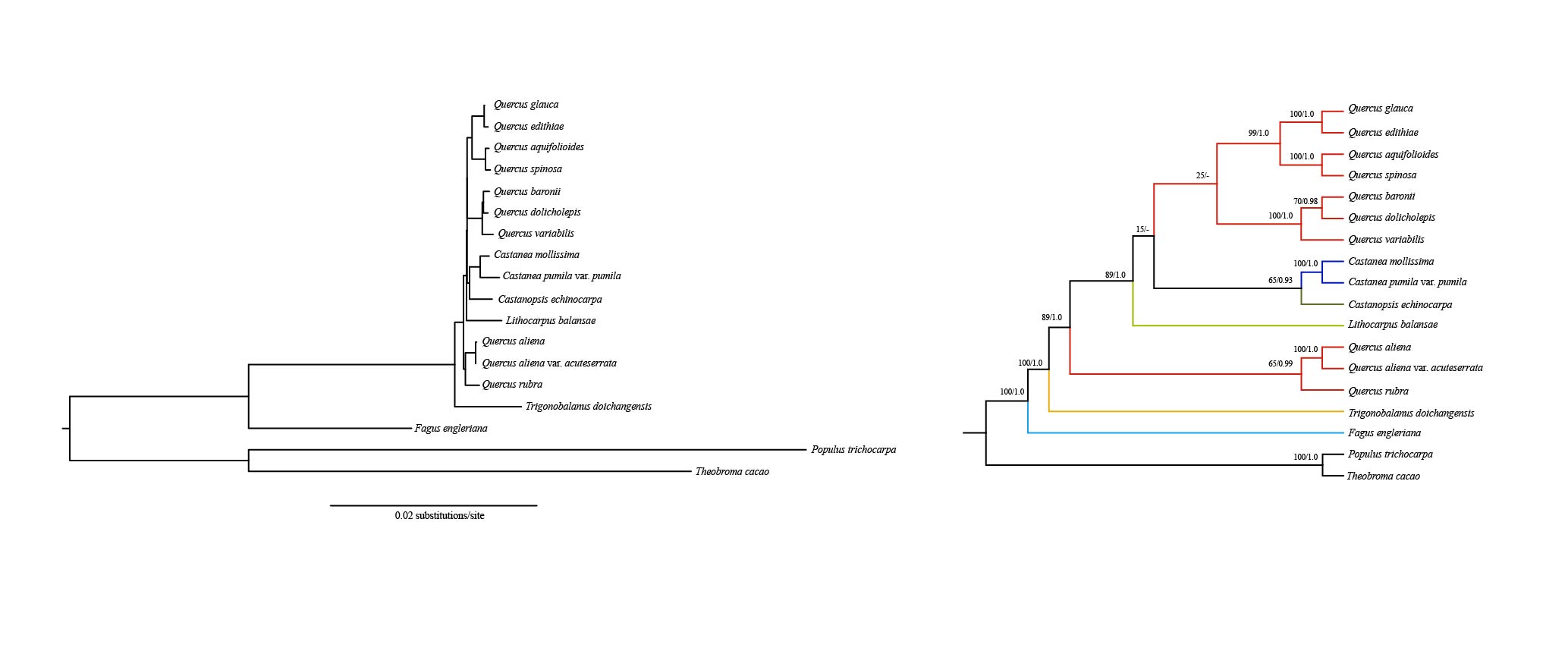

Supplement: Figure S4 — Fagaceae phylogeny based on ML and BI analyses of genes belonging to photosynthetic metabolism functional category. ML topology shown with bootstrap support values and posterior probability values listed at each node. Dash denotes nodes contradicted by the BI trees with posterior probability values < 0.50. [file Image4.JPEG]

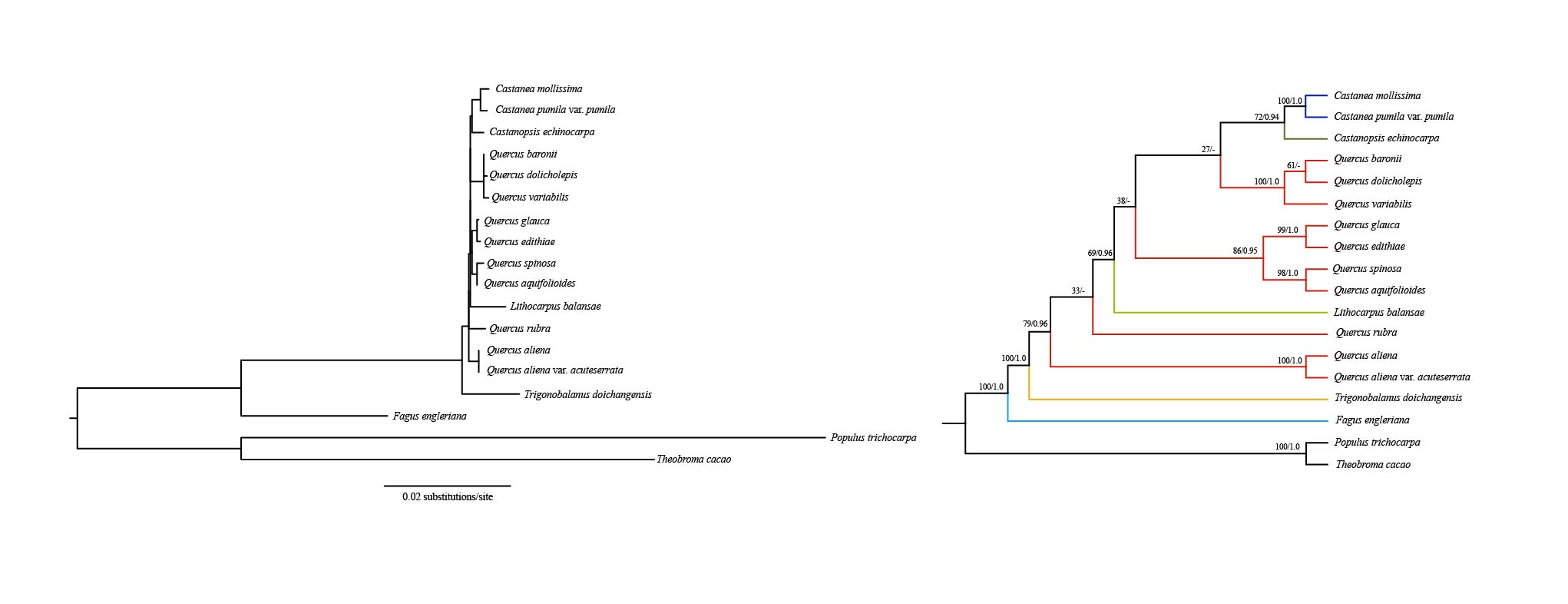

Supplement: Figure S5 — Fagaceae phylogeny based on ML and BI analyses of genes belonging to miscellaneous functional category. ML topology shown with bootstrap support values and posterior probability values listed at each node. Dash denotes nodes contradicted by the BI trees with posterior probability values < 0.50. [file Image5.JPEG]

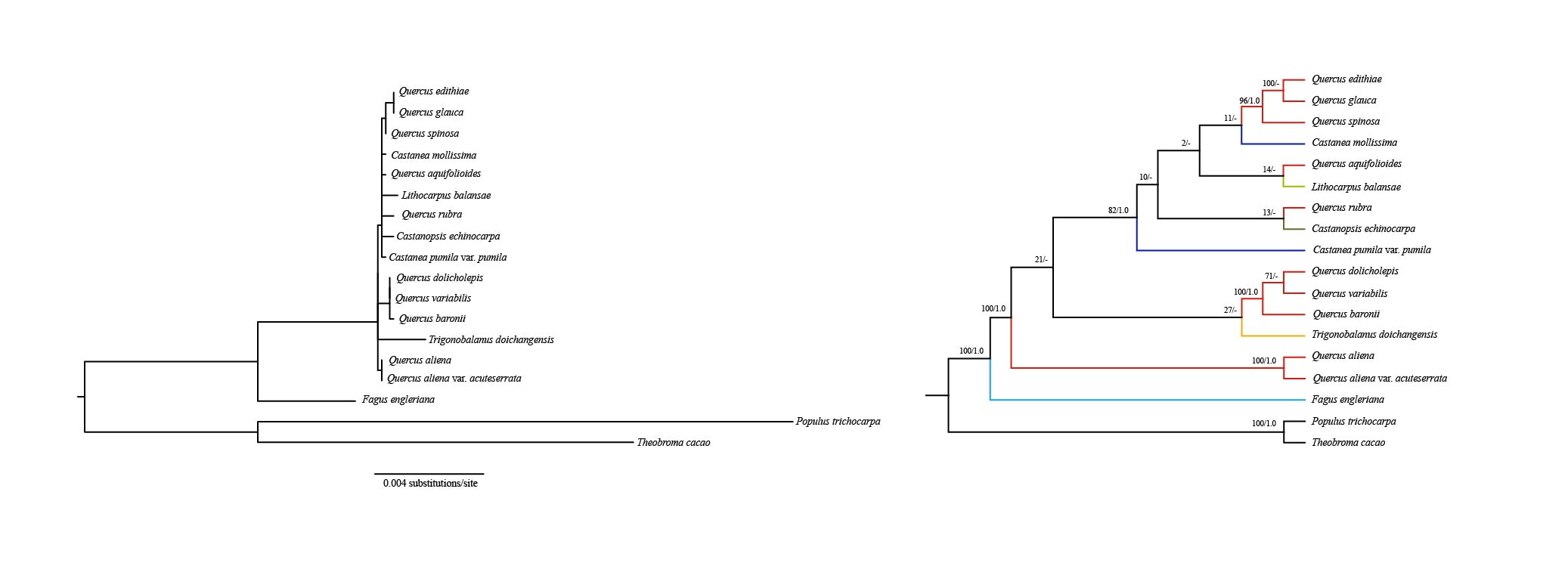

Supplement: Figure S6 — Fagaceae phylogeny based on ML and BI analyses of genes belonging to unknown functional category. ML topology shown with bootstrap support values and posterior probability values listed at each node. Dash denotes nodes contradicted by the BI trees with posterior probability values < 0.50. [file Image6.JPEG]
